# Incidence, Treatment and Outcome of Patients with Retroperitoneal Soft-Tissue Sarcoma in Switzerland 2005–2015: A Population-Based Analysis

**DOI:** 10.1007/s00268-021-06374-z

**Published:** 2021-11-09

**Authors:** Johanna C. F. Willburger, Marco von Strauss, Caspar J. Peterson, Tracy R. Glass, Christoph Kettelhack

**Affiliations:** 1 Department of Visceral Surgery, University Center for Gastrointestinal and Liver Diseases, St. Claraspital and University Hospital Basel, Clarunis, 4002 Basel, Switzerland; 2 Center for Bone and Soft Tissue Sarcoma, University Hospital Basel, Spitalstrasse 21, 4031 Basel, Switzerland; 3 Department of Epidemiology and Public Health, Swiss Tropical and Public Health Institute, P.O. Box, 4002 Basel, Switzerland; 4 Department of General Surgery, Kantonsspital Graubünden, Loëstrasse 170, 7000 Chur, Switzerland

## Abstract

**Background:**

Reports on the epidemiology and mortality of retroperitoneal soft tissue sarcoma (RSTS) in Switzerland are scarce. This study investigates the incidence and outcomes of surgically treated RSTS inpatients in Switzerland depending on the hospital type and size.

**Methods:**

Data from the Swiss Federal Statistical Office were used to conduct a retrospective analysis of all RSTS inpatients and hospitalizations in Switzerland between 2005 and 2015. RSTS was identified by the code C48.x of the International Classification of Diseases (ICD-10). Sarcoma centers were identified by the annual total number of sarcoma patients (> 50 patients/year). The analysis of yearly incidence, age distribution as well as in-hospital complication and mortality was performed for non- and surgical-treated patients. A centralization of treating sarcoma patients was analyzed by the trend of hospitalizations in sarcoma centers and high-volume hospitals.

**Results:**

During 2005–2015, 2.801 hospitalizations (1651 patients) were admitted to Swiss hospitals with the primary diagnosis of a RSTS. The yearly number of RSTS patients and the incidence (1.91/100.000) stayed constant within these 11 years. There were five sarcoma centers. We saw a clear trend of RSTS patients being treated (especially surgically) in centers over the 11 years. The complication rate of surgical-treated patients was higher in sarcoma centers (55% vs. 40%), though the overall mortality rate was lower (3.2% vs. 9.1%).

**Conclusion:**

Centralization of RSTS treatment to certified sarcoma centers leads to a lower overall mortality rate and thus is highly recommended.

## Introduction

In Europe, the incidence of sarcoma is 5.6 per 100,000 people per year. Sarcomas are typically divided into two major groups: soft tissue sarcomas (84%) and bone sarcomas (14%). For soft tissue sarcomas of any anatomic localization, the incidence is 4–5 per 100.000 persons per year [1, 2]. Sarcomas account for approximately one percent of all malignant tumors in adults, and for up to 15% of malignant tumors in children (0–4 years). [3, 4]

International studies have shown that treatment of sarcoma in a certified sarcoma center (SC), as opposed to a non-certified clinic, results in better patient outcomes (i.e., fewer re-operations, more tumor-free resection margins) [1, 5–7]. In certified SC, well-established interdisciplinary teams of surgical sarcoma specialists, plastic surgeons, oncologists, radiologists and radiation oncologists can provide standardized treatment pathways for these otherwise rare conditions [8–10].

In fact, the ‘Trans-Atlantic Retroperitoneal Sarcoma Working Group’ recommends treatment of retroperitoneal soft tissue sarcomas (RSTS), which often require complex multi-visceral resections, be planned and performed only in dedicated SC, where multidisciplinary sarcoma teams and tumor conferences are available [11].

Our study focuses on the national incidence and outcome of patients with RSTS treated in Switzerland. Epidemiological data on the incidence and outcome of this disease are currently lacking since treatment of soft tissue sarcomas is not centralized in Switzerland. Based on a national cohort during an eleven-year period between 2005 and 2015, our aim was to analyze the caseload of RSTS patients and their outcomes depending on the hospital type. We hypothesized that hospitalization in a SC or high-volume hospital (HVH) correlates with a better outcome for patients with RSTS. The outcome was assessed by the rate of post-surgical complications and mortality.

## Material and methods

The data analyzed in this study was obtained from the ‘Swiss Federal Statistical Office’ (Bundesamt für Statistik [BFS]). Swiss law requires all Swiss hospitals to report data for all inpatient cases to the BFS. These reports include sociodemographic data, administrative data and medical data such as the ‘International Statistical Classification of Diseases and Related Health Problems’, 10^th^ revision (ICD-10) codes for all diagnoses, details about the treatments received, as well as several outcome measures. The anonymized data sets are available for research and audit purposes after a peer-reviewed application process. At the time of our application, data were available for the years 2005–2015. After gaining access to the data sets, we screened for patients with RSTS.

For all patients hospitalized with RSTS between 2005 and 2015, we extracted demographic data, number and frequency of hospitalizations, type of admission (elective vs. emergency), hospital type (volume-size, SC status), treatment (surgical vs. non-surgical), complications and mortality. The annual incidence of RSTS in Switzerland was calculated using Switzerland’s population data from the BFS [12].

RSTS have no specific code in the ICD-10. Therefore, we decided to use the codes C48.x (malignant neoplasm of retroperitoneum and peritoneum) [13, 14].

To determine whether a hospital qualifies as a SC, we counted the annual total number of sarcoma patients and hospitalizations for the following ICD-10 codes listed in Table 1.

**Table 1:** ICD-10 codes for different types and locations of sarcoma to define a SC (included non-surgical-treated patients)

C40	Malignant neoplasm of bone and articular cartilage of limbs
C41	Malignant neoplasm of bone and articular cartilage of other and unspecified sites
C47	Malignant neoplasm of peripheral nerves and autonomic nervous system
C48	Malignant neoplasm of retroperitoneum and peritoneum
C49	Malignant neoplasm of other connective and soft tissue

The Swiss classification of surgical procedures (Schweizer Operationsklassifikation [CHOP]) code, which categorizes and codes surgical treatments and diagnostics [12], was used to distinguish between surgical and non-surgical treatment.

Postoperative complications were identified by ICD-10 codes [13, 14] that were found in the patient diagnoses reports (Table 2).

**Table 2:** Applied ICD-10 codes of complications

D 62	Acute posthemorrhagic anemia
J 93	Pneumothorax
J 95	Postprocedural respiratory disorder, unspecified
K 43	Hernia ventralis
K 44	Hernia diaphragmatica
K 56	Paralytic ileus and mechanical ileus without hernia
K 65	Peritonitis
K 91	Postprocedural disorder of digestive system, unspecified
N 99	Postprocedural renal failure, unspecified
T 81	Other complications of procedures, not elsewhere classified
T 88	Other specified complications of surgical and medical care, not elsewhere classified
Y 84	Other medical procedures as the cause of abnormal reaction of the patient, or of later complication, without mention of misadventure at the time of the procedure

In case of emergency admission, the codes K56 and K65 were not counted toward complications because they could have been the reason for the hospitalization rather than a complication. The true number of emergency hospitalizations in RSTS patients cannot be established with this limitation. Therefore, we concentrated on the total number of cases and on elective hospitalizations.

We used the Charlson comorbidity index (CCI) [15] to evaluate the patients’ condition and interpret the mortality rate in different types of hospitals (SC, high or low volume hospitals LVH).

To compare mortality rates, we concentrated on the *surgically* treated patients, the *elective* hospitalizations and the *overall* mortality (elective and emergency hospitalization). The CCI and the patients’ age was used to include the patients’ condition into our interpretations.

### Sarcoma centers and high- or low-volume hospitals

Hospitals treating more than 7,500 patients annually for at least one year between 2005 and 2015 were considered HVH, as opposed to LVH with less than 7,500 hospitalizations per year. The threshold of 7,500 patients is set by the BFS. HVH corresponded mostly to the larger medical centers in Switzerland. LVH corresponded mostly to Swiss regional hospitals.

Hospitals treating a minimum of 50 patients (non-surgically) per year for at least one year between 2005 and 2015 for any type and location of sarcoma were considered SC.

### Statistical analysis

Data were described using appropriate descriptive statistics, mean, standard deviation, mean and interquartile range for continuous variables and frequency and percentages for categorical variables. Trends in hospitalization rates and comparison between binary outcomes, such as complications and mortality, were made using Chi-square tests to calculate p values. A p value of < 0.05 was deemed statistically significant.

All analyses were done using Stata version 15.1 (StataCorp LP, College Station, TX, USA).

Concerning our results, we differentiate between:

- Patient: one individual with a diagnosis of RSTS
- Hospitalization: number of occasions concerning a RSTS.

We checked for data irregularities and duplication and removed corresponding cases.

## Results

### Demographic Results

A total of 1651 patients were treated for RSTS (ICD-10 code C48.x) in Swiss hospitals between 2005 and 2015 for a total of 2801 hospitalizations (Table 3).

**Table 3:** Patient demographics, hospital data and sarcoma type

	Total number	%
Hospitalizations	2801	
Patients	1651	
*Age*		
Mean Age	66y	
Mean length of hospitalization [range]	10d [0-283d]	
*Sex*		
Male	554	34
Female	1097	66
Median CCI (IQR) [range]	2 (2–8) [2–44]	
Mean CCI (SD)	5.3 (5.3)	
*Nationality*		
Swiss	1421	86
Non-Swiss	230	14
*Language region*		
German	1232	75
French	348	21
Italian	71	4
*Sarcoma Typification C48.x*		
C48.0 Retroperitoneum	606	37
C48.1 Specified parts of peritoneum	435	26
C48.2 Peritoneum, unspecified	447	27
C48.8 Overlapping lesion of retroperitoneum and peritoneum	163	10

Table 4 shows the number of inpatient RSTS patients in Switzerland, as well as the incidence of inpatients with a RSTS disease calculated with the yearly Swiss population. The average incidence from 2005 to 2015 was 1.91/100.000.

**Table 4:** Demographic overview of sarcoma and RSTS patients in Switzerland from 2005–2015

Year	RSTS patients	Total sarcoma patients	RSTS incidence/100.000 in Switzerland
2005	148	591	1.98
2006	160	575	2.13
2007	133	552	1.75
2008	162	516	2.10
2009	161	771	2.07
2010	145	807	1.84
2011	124	847	1.56
2012	152	824	1.89
2013	151	897	1.86
2014	146	872	1.77
2015	169	935	2.03

The total number of sarcoma patients increased from 591 in 2005 to 935 patients in 2015, whereas the number of C48.x patients remained constant. We were unable to determine the reason behind the sudden increase in total sarcoma patients from 2008 to 2009.

### Patient Distribution According to the Hospital Size and Sarcoma Center Criteria

We identified 176 hospitals with at least one documented hospitalization for soft tissue sarcoma between 2005 and 2015. Out of the 176, 73 (41%) were considered HVH and five were SC. SC is made up 3% of all 176 listed hospitals and 7% of HVH. There was no LHV with SC status.

**Fig. 1:**
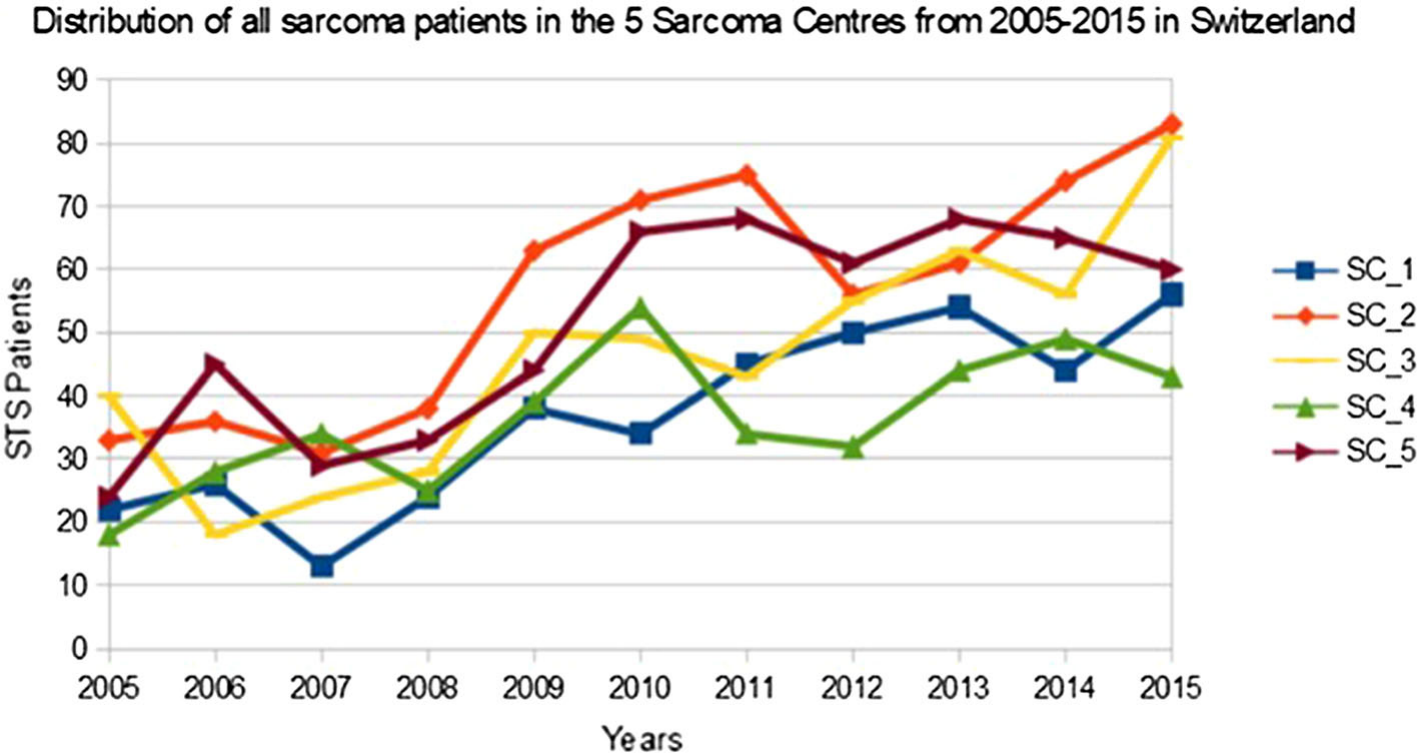
Annual number of sarcoma patients per year in the five SC (labeled as SC_1–5) from 2005 to 2015 in Switzerland

The five hospitals identified as SC have fulfilled the SC criteria for 1 to 7 years between 2005 and 2015 (Fig. 1), but all were considered HVH during the entire 11-year period.

### Retroperitoneal Soft Tissue Sarcoma

We found a significant increase in hospitalizations in HVH from 2005 to 2015, with 68% of all RSTS patients being hospitalized in HVH in 2005 and 82% in 2015 (*p* = 0.005).

**Fig. 2:**
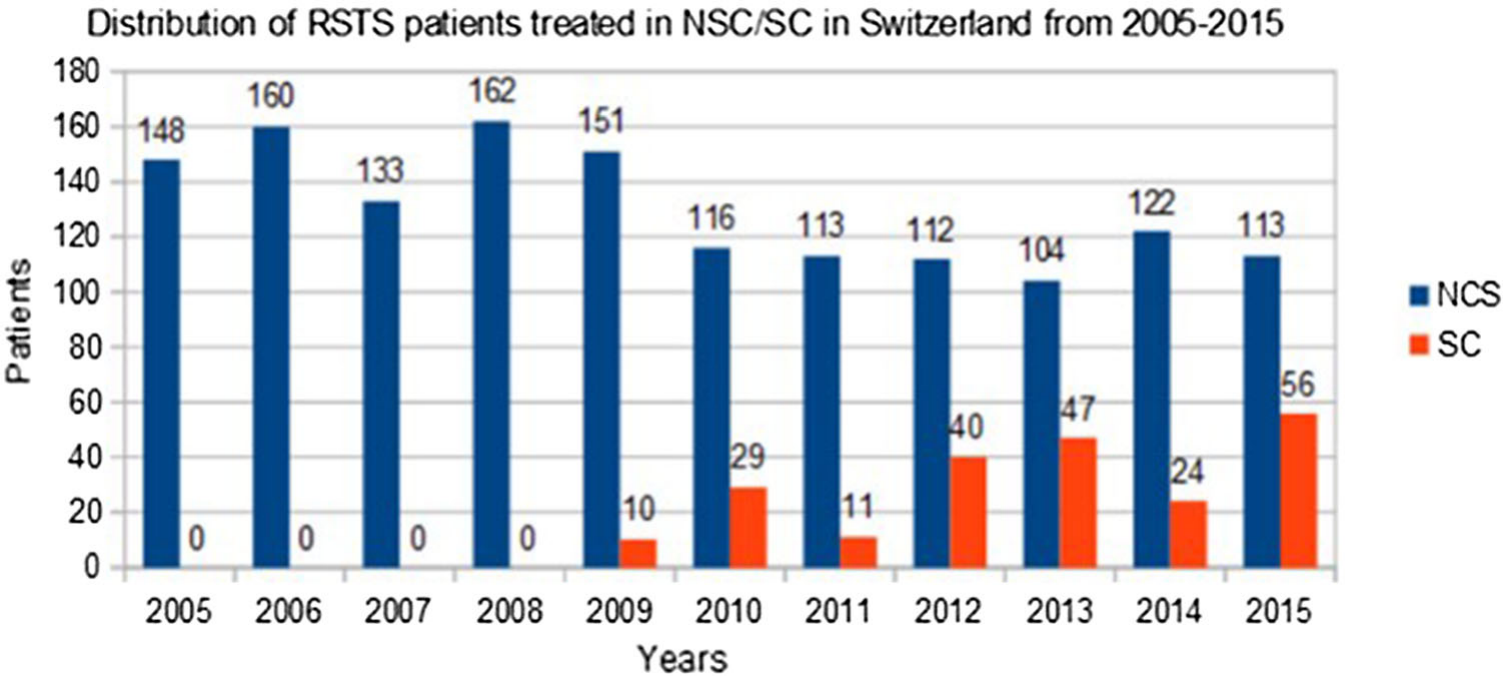
Distribution of RSTS patients depending on their treatment in SC vs. NSC in Switzerland between 2005 and 2015

A similar trend was evident concerning the proportion of RSTS patients being treated in SCs starting at 0% in 2005, climbing to 6% in 2009, and peaking with 33% in 2015 (*p* < 0.001) (Fig. 2).

We observed a higher percentage of surgical treatment during hospitalization in HVH compared to LVH (53% versus 34%, *p* < 0.001). Similarly, the percentage of surgical treatment during hospitalization was higher in SC compared to non-sarcoma center (NCS) (56% versus 46%, *p* < 0.001). A detailed overview is given in Table 5.

**Table 5:** Summary of the total number and percentage of patients and hospitalizations with retroperitoneal sarcoma with surgical treatment according to hospital size and NSC/SC status

		Patients	%	Hospitalizations	%
		n		n	
Total		1651		2801	
Surgical treatment		1008	61	1328	47
Volume	HVH	1200	73	2027	72
	Surgical treatment	808	67	1065	53
	LVH	451	27	774	28
	Surgical treatment	200	44	263	34
SC status	SC	220	14	410	15
	Surgical treatment	164	75	228	56
	NSC	1431	86	2391	85
	Surgical treatment	844	59	1100	46

### Complications

Among all *elective hospitalizations* (non-surgically and surgically treated patients) for a RSTS, we found a total of 757 complications (0 to 8 complications per hospitalization), leading to an overall complication rate of 27%.

The comparison of complication rates of *elective* RSTS patients being *surgically* treated in SC vs. NSC is given in Table 6.

**Table 6:** Complication number/rate of elective hospitalizations after surgical treatment depending on hospital type

In *elective hospitalization* (n = 1328)	Complication n	Complication %
Total complication after surgery	562/1328	42%
SC (n = 228)	126 /228	55%
NSC (n = 1100)	436 /1100	40%
HVH (n = 1065)	483 /1065	45%
LVH (n = 263)	79 /263	30%

The higher complication rate in SC and HVH compared to NSC and LVH was significant (*p* < 0.001).

### Mortality

Overall RSTS patients, we found an in-hospital mortality rate of 16% (272 recorded deaths in 11 years).

We compared the average age and CCI depending on the hospital type/volume (Table 7).

**Table 7:** Summary of the total number and in elective hospitalized surgical-treated RSTS patients depending on the hospital type in respect of the CCI and age

Hospital type	Total mortality rate of surgical-treated RSTS patients (elective and emergency)	Mortality of surgical-treated RSTS patients in elective hospitalization	Median CCI	Median Age
Total mortality after surgery	5.8% (58/1008)	3.5% (27/774)		
SC	2.4% (4/164)	1.4% (2/146)	5.7	62
NSC	6.4% (54/844)	4.0% (25/628)	5.2	69
HVH	5.1% (41/808)	3.3% (21/632)	5.6	66
LVH	8.5% (17/200)	4.2% (6/142)	4.6	69

The mortality rate in emergency hospitalization was significantly higher in SC (11.1%; 2/18 patients), NCS (13.4%; 29/216), HVH (11.4%; 20/176) and LVH (19%; 11/58) compared to elective hospitalizations (compare Table 3).

The difference in mortality rate between surgical treatment after emergency and elective hospitalization is significantly lower in SC than in NSC. We found same results when comparing HVH to LVH.

Based on these results, we conclude that the treatment of RSTS patients in SC and HVH can be highly recommended, despite the higher rate of complications in SC and HVH, which are though mostly attributed to the greater complexity of the individual patient. Therefore, treatment of RSTS patients in SC/HVH is highly recommended.

## Discussion

RSTS is a rare disease whose treatment necessitates a great amount of experience provided by an interdisciplinary team. These conditions are given in certified SC [1]. Our retrospective study analyzes patient demographics and treating hospitals from 2005 to 2015 in Switzerland. Specifically, we analyzed the distribution of RSTS patients in this 11-year period depending on the hospital type (SC versus NSC) and volume (HVH or LVH) these patients were treated in. In order to evaluate the complications and mortality rate, we differentiated between surgically and non-surgically treated RSTS patients.

### Demographic Results

Overall, our Swiss data are in line with international data concerning patient demographics (age and sex distribution, incidence [2]), which makes our results reliable [4]. A total of 1651 patients, accounting for 2801 hospitalizations, were treated for a RSTS, defined as ICD-10 code C48.x, in Switzerland between 2005 and 2015. Within this period, we noticed a slight rise in number of first hospitalizations, which is in accordance with the population growth in Switzerland. Therefore, the average incidence of 1.9/100.000 patients per year remained constant over the 11-year period and is comparable to the incidence rates of international studies [16, 17].

The average length of hospitalization was 10 days. The time spread from 0 (= less than 24 h, overnight) to 283 days shows the variation of severity of the disease and complexity of the treatment.

A total of 66% of the overall 1651 RSTS patients were female and 34% were male, which are not in accordance with the current literature. Most epidemic data show an equal gender distribution [2, 5], even though a slight relationship between female hormones and the development of a soft tissue sarcoma has been mentioned [18].

The median age of our patients was 67 years, with two age peaks. The first and smaller one is in children from 0 to 4 years of age (19 patients) and the second in elderly people from 70 to 74 years of age (244 patients). This age distribution is also described in other epidemic studies [4, 19, 20].

### SC and HVH: Distribution and Treatment

Between 2005 and 2015, five hospitals in Switzerland qualified as SC, defined as hospitals that treated more than 50 inpatients for any kind of sarcoma [21] for at least 1 of the 11 years. Within this period, the number of sarcoma inpatients in these SC varied between 13 and 83. We noticed a distinct increase in recorded sarcoma inpatients between 2008 and 2009. Therefore, the conditions to qualify as a SC were fulfilled by most of the mentioned hospitals in 2009. We checked for differences between the 2008 and 2009 version of ICD-10 codes without finding any difference. However, the absolute number of C48.x patients was constant over the years.

We identified a trend toward RSTS patients being increasingly treated in SC over the observed 11-year period, as recommended in international guidelines [1]. While no patient treatment in a SC was registered between 2005 and 2008, already 6% and 33% of RSTS patients were treated in SC in 2009 and 2015, respectively. A similar trend was evident for the distribution of treatment conducted in HVH, which increased from 68% in 2005 to 82% in 2015.

Every hospital that qualified as SC for at least 1 year between 2005 and 2015 also qualified as HVH. We had expected this overlap since a SC necessitates a multi-disciplinary team to organize qualified treatment. This complex setting is rarely available in LVH [22, 23].

To distinguish between the quality of treatment in SC or NSC and HVH or LVH, we analyzed surgically and non-surgically treated patients separately.

We observed a significantly higher rate of surgically treated patients (and hospitalizations) in HVH and SC, than in LVH and NSC. These data suggest that surgical treatment and the overall management of sarcoma patients are increasingly taking place in SC [22]. However, up to 2015, this trend toward centralization is still far from the desired state.

### Complications

In elective hospitalization, we have a higher complication rate in surgically treated patients in SC than in NSC (55% versus 40%). The same trend was evident concerning HVH and LVH (45% versus 30%). We assume that the patients treated in SC or HVH are the more severe cases needing more challenging treatment associated with a higher rate of complications. However, the present data allow only a limited interpretation of these numbers. We have no information if complications in emergency hospitalizations linked the patient’s sarcoma disease or any non-surgical treatment.

The greater complexity of the patients treated in SC and HVH may be reflected by the average CCI score. In case of a hospitalization in a SC, the CCI was 0.5 points higher than in NSC (5.7 vs. 5.2), and in HVH, the CCI was even 1.0 points higher than in LVH (5.6 vs. 4.6). However, these differences were not significant.

### Mortality

We were able to show that RSTS patients who were surgically treated in a SC had a significantly lower risk of death than those treated in a NSC, even though there were overall more surgical treatments, and the condition of the patient were more severe. The higher mortality rates in low-volume hospitals might be explained by higher rates of palliative care sarcoma patients. However, this remains speculative. Unfortunately, we have no information about the rate of palliative treatment in different hospitals.

### Limitations

There are several limitations to this study. First, the ICD-10 code C48.x, which was used to identify RSTS, is not specifically defined as “sarcoma” but rather as “malignoma of the retroperitoneum” in the ICD-10. This may have led to the inclusion of retroperitoneal malignomas other than sarcomas in our analysis. However, we used the ICD-10 code C48.x to identify RSTS patients, as other international studies did as well [17, 24, 25]. The most frequent retroperitoneal malignancies like lymphoma (C83-86, C 91) or gynecological tumors (C53-58) have their own respective ICD-10 codes and therefore can be excluded if we assume initial coding was done correctly [14]. There was no data on macro- or microscopic (R1/R2 Resection) appearance of the tumor, which would make an essential difference in interpreting the extent of surgery and complication/mortality rates.

Second, we were only able to identify the inpatient RSTS patients. Therefore, our results are just an approximation. The creation of a national sarcoma network to collect all data of RSTS patients, including epidemiological information as well as treatment and histological information, would greatly facilitate the interpretation of data [2, 26].

Third, our retrospective analysis is based on data collected between 2005 and 2015. This means RSTS patients first treated before 2005 were counted as new cases instead of re-hospitalizations, and patients re-hospitalized after 2015 were not included in this study.

We assume that applying a volume cut-off of 7,500 patients being hospitalized per year to qualify as a HVH might render a comparison with international data more difficult, since the volume cut-off used in larger epidemiological studies is usually much higher. Yet, we decided to adhere to the cut-off defined by the Swiss Federal Statistical Office for our Switzerland-based study.

## Conclusion

Based on these results, we conclude that a centralization of complex sarcoma surgery is accompanied by a lower mortality rate. This is mainly underlined by the lower mortality rate after elective surgical treatment of these patients. The fact that we saw a higher rate of complications in SC and HVH was observed is possibly attributed to the greater complexity of the surgical intervention and the individual patient (higher CCI scores).

Therefore, treatment of RSTS patients in SC/HVH should be supported by official certification requirements.

## Abbreviations

BFS: Bundesamt für Statistik = Swiss Federal Statistical Office
CCI: Charlson comorbidity index
ICD: International Statistical Classification of Diseases and Related Health Problems
HVH: High-volume hospital
IQR: Interquartile Range
LVH: Low-volume hospital
NSC: No sarcoma center
RPS: Retroperitoneal sarcoma
RSTS: Retroperitoneal soft tissue sarcoma
SC: Sarcoma center
SFSO: Swiss Federal Statistical Office
